# Human Germline: A New Research Frontier

**DOI:** 10.1016/j.stemcr.2015.04.014

**Published:** 2015-05-28

**Authors:** M. Azim Surani

**Affiliations:** 1 Wellcome Trust Cancer Research UK Gurdon Institute, University of Cambridge, Tennis Court Road, Cambridge CB2 1QN, UK; 2 Department of Physiology, Development and Neuroscience, University of Cambridge, Downing Street, Cambridge CB2 3EG, UK; 3 Wellcome Trust-Medical Research Council Stem Cell Institute, University of Cambridge, Tennis Court Road, Cambridge CB2 3EG, UK

## Abstract

We recently elucidated the mechanism of human primordial germ cell (hPGC) specification and resetting of the epigenome for totipotency. The regulators of hPGC specification also initiate resetting of the epigenome, leading to a comprehensive erasure of DNA methylation, erasure of imprints and X reactivation in early hPGCs in vivo. These studies reveal differences with the mouse model, which are probably due to differences in the regulation of human pluripotency, and in postimplantation development at gastrulation, which indicates the importance of non-rodent models for investigations. Within the extreme hypomethylated environment of the early human germline are loci that are resistant to DNA demethylation, with subsequent predominant expression in neural cells. These loci provide a model for studies on the mechanism of transgenerational epigenetic inheritance, and their response to environmental factors. Such epigenetic mechanism of inheritance could potentially provide greater phenotypic plasticity, with significant consequences for human development and disease.

## Main Text

### Germline: The Immortal Lineage

A primary role of germline is to generate the totipotent state, which precedes establishment of pluripotency during preimplantation development (Hayashi and Surani, 2009; Leitch et al., 2013). With totipotency, human germline not only gives rise to a new organism, but also theoretically at least, to an endless series of generations. Thus, germ cell lineage is considered “immortal,” unlike somatic cells that perish with each individual. Germline transmits genetic as well as epigenetic information to subsequent generations. To accomplish this significant role, the germline epigenome undergoes comprehensive and unprecedented chromatin modifications, and global erasure of DNA methylation (Hackett et al., 2013; Kagiwada et al., 2013; Seisenberger et al., 2012). This process will also ensure erasure of epimutations. Without such erasure, there would be progressive accumulation of epimutations, which would compromise germline functions and survival of the species.

DNA methylation is also a key mechanism for the repression of transposable elements (TEs). The global erasure of DNA methylation therefore creates conditions for the activation of TEs and their transpositions (Zamudio and Bourc’his, 2010; Tang et al., 2015). These repetitive elements make up more than half of the mammalian genome, indicating that neither the invasion of our genome by these foreign elements, nor their expansion once acquired can be completely restrained. The comprehensive erasure of DNA methylation creates a key battleground between TEs and host defense mechanisms, resulting in an arms race to regulate their activity. Transposition events have the potential for inducing mutations; however, not all of these will have deleterious consequences. The TEs have also been crucial for mammalian evolution; some have been co-opted for important functions to regulate mammalian development (Gifford et al., 2013).

Mammalian germline also generates critical epigenetic information for totipotency and development through imprinted genes. Expression of these genes is strictly dependent on their parental origin, which explains why both male and female genomes are essential for mammalian development. Imprints are erased and re-initiated in the germline; following fertilization, they are subsequently detected as robust and heritable parent-of-origin-dependent DNA methylation marks in embryos that persist into adulthood. Imprinted genes provide reciprocal epigenetic information in parental genomes, which results in functional differences between parental genomes during development. Thus, whereas the parental genomes contribute equivalent genetic information to the zygote, the epigenetic information strictly depends on their parental origin. Parental imprints are first erased in primordial germ cells (PGCs) and then re-established appropriately during every germline cycle, and not passed on transgenerationally. Inheritance of epigenetic information through imprinting is a highly regulated process with clearly defined mechanism for erasure and re-initiation.

This epigenetic information transmitted from germline via imprinted genes, differs from the epigenetic information that is apparently acquired in response to diverse environmental factors, and transmitted through the germline. The mechanistic basis for how such epigenetic information might be acquired and transmitted either inter- or transgenerationally is unclear (Radford et al., 2014; Heard and Martienssen, 2014), which remains a major question for mammalian germline biology. The consequences of such epigenetic inheritance in regulating phenotypic traits and any potential role during mammalian evolution also remain to be elucidated.

### Pangenesis, Gemmules, Germ Plasm, and Mobile RNAs

Darwin proposed pangenesis in 1868 as a “provisional” hypothesis of heredity. He proposed that organs produce “gemmules,” which contain information on the performance of each organ in the body (Darwin, 1868). These gemmules are than passed on to sperm and eggs, and in this way, information from somatic tissues is gathered and transmitted to the next generation. Some recent reports on environmentally induced epigenetic changes and their apparent transgenerational inheritance conform to the idea of pangenesis, which has overtones of Lamarckian inheritance of acquired characters (reviewed by Heard and Martienssen, 2014). Although this is unlikely, it does not entirely negate a degree of phenotypic plasticity that could be induced by environmental factors, albeit the mechanistic basis for the inheritance of such information through the mammalian germline is difficult to envisage. Non-coding RNAs, might be thought of as gemmules, in particular, mobile RNAs in plants and nematodes have been proposed as agents for transmission of information from cell to cell, and potentially through the germline (Sarkies and Miska, 2014). However, in mammals, the germline is set aside during early postimplantation development, which poses additional barriers to be overcome for such transmission from soma to germline.

The idea of a barrier separating germline from soma was proposed by August Weissmann, who in 1889 proposed the concept of germ plasm. Accordingly, only cells that inherit germ plasm acquire germ cell fate, and the remaining cells acquire somatic fates. Furthermore, only the cells inheriting germ plasm during each generation transmit genetic information to the next generation, excluding somatic cells from any such role. A strict interpretation of this idea is that germ cells do not carry information from somatic cells as far as inheritance is concerned. This is sometimes referred to as Weissman’s Barrier, which challenges the Lamarckian idea of inheritance of acquired characters. With the advent of induced pluripotent stem cells (iPSC) however, it is possible to generate human primordial germ cells from adult somatic cells via iPSC (Irie et al., 2015), which to some extent breaks the Weissman’s Barrier. It is clearly important to resolve the issue of environmentally induced transmission of epigenetic information through the human germline, which apparently has phenotypic consequences. To address this question, it is first essential to know how the human germ cell lineage is established, and gain knowledge of how the germline epigenome is reset. Our recent work has been directed at addressing some of these fundamental questions concerning the human germline (Irie et al., 2015; Tang et al., 2015).

### Specification of Human Primordial Germ Cells

First, it is important to elucidate the mechanism of human PGC specification, the precursors of sperm and eggs. PGC specification in mammals does not depend on the inheritance of germ plasm, but is induced by signaling molecules during early postimplantation development (De Fellici, 2013). Indeed, some evidence indicates that all pluripotent cells in blastocysts and all pluripotent embryonic stem cells (ESCs) are potential PGCs. Unlike in some organisms, mammalian germ cells are not allocated early in development. Lawson and Hage (1994) studied the origin of PGCs in mouse embryos and observed them through early postimplantation development to the establishment of founder population of PGCs in mice, which are induced by BMP4 (Lawson et al., 1999). Importantly, genetic studies identified key transcription factors that are induced by BMP4, which play an essential role in germ cell fate determination. These factors are also important for initiating a program for resetting the germline epigenome (Hayashi and Surani, 2009).

We first established the genetic basis of mammalian PGC specification in mice using a single cell transcriptome analysis, which led to the identification of *Prdm1* (encoding BLIMP1) as a key regulator of PGCs (Saitou et al., 2002; Ohinata et al., 2005; Hayashi et al., 2007). A key role of BLIMP1 is to repress somatic fate in the postimplantation epiblast cells from which PGCs are recruited. BLIMP1 mutant cells fail to undergo specification as PGCs and show expression of somatic genes. The use of BLIMP1 mutant cells also led to the identification of PRDM14, which has a significant role in regulating pluripotency and during specification of PGCs (Magnúsdóttir et al., 2013; Nakaki et al., 2013). A third critical gene *Tfap2c* (encoding AP2G), is a direct target of BLIMP1 (Magnúsdóttir et al., 2013). These regulators constitute a tripartite genetic network for mouse PGC specification, which are necessary and sufficient for mouse PGC specification. They act combinatorially by binding to targets to regulate three key functions: suppression of somatic fate, regulation of germ cell program, and the epigenetic program. Genetic studies confirmed that a mutation in BLIMP1 or PRDM14 abrogates PGC fate in vivo. An in vitro method allows development of PGC-like cells from naive pluripotent stem cells (ESCs), with a potential to develop into viable gametes (Hayashi et al., 2011). PGCs can be induced by cytokines or directly by the three transcription factors in vitro without cytokines (Magnúsdóttir et al., 2013; Nakaki et al., 2013).

Our recent work has focused on the mechanism of human PGC specification, which occurs during week 2 of gestation, and therefore cannot be directly investigated in early human embryos. Based on mouse studies, hESCs could be used to examine induction of PGC-like fate in vitro although the mouse model does not work with human ESCs (Irie et al., 2015). Furthermore, hESCs cultured in conventional culture conditions have a very limited potential for hPGCLC specification. However, we found that hESCs maintained in “4i” culture conditions acquire and maintain high competence for hPGCLC-like fate. hPGC-like cells could be induced very efficiently in these competent hESCs by cytokines containing BMP2/BMP4 (Gafni et al., 2013; Irie et al., 2015). Notably, hESCs lost competence for hPGCLC fate when they were returned to conventional culture conditions. Transcriptome analysis of hPGCLC and comparison with authentic in vivo wk7-wk9 hPGCs, and a seminoma cell line showed that they shared expression of key PGC genes, among which were SOX17 and a cell surface marker CD38. Further analysis of the sequence of gene expression during hPGCLC specification revealed that SOX17 expression is detected first, followed by BLIMP1. Notably, SOX17 has no role in the specification of mouse PGCs. The role of PRDM14 in hPGC specification also remains unclear, in contrast to its pivotal role in mouse PGC specification. There are other genes whose functions remain to be elucidated, including GATA4 and TEAD4. Notably, hPGCLCs and hPGCs do not show expression of SOX2; however, TFCP2L1 and KLF4 are detected in hPGCs. There is no expression of KLF4 in mouse PGCLC; however, whereas SRBB1 expression is detected in mouse PGCs, expression of this gene is undetectable in hPGCs (Irie et al., 2015; Tang et al., 2015). This shows that there are significant differences between mouse and human PGC specification. The differences in culture conditions that confer competence for PGC fate in ESCs also apparently differ between mouse and human. Further work is needed to clarify the precise molecular basis for how competence for PGC fate is acquired and lost in the two species. Apart from the differences in the regulation of human and mouse pluripotent states (Takashima et al., 2014), there are also differences in their postimplantation development; postimplantation embryos in rodents develop as egg cylinders, whereas human and many or most other mammalian embryos develop as bilaminar discs. This could affect the mechanism that confers competence for PGCs, and other early cell fate decisions. It is important therefore to explore non-rodent mammalian models for early postimplantation development and gastrulation.

Studies also show that the response to SOX17 during hPGCLC is dose dependent because reduced numbers of hPGCLCs are detected in SOX17 heterozygous hESCs. Loss of SOX17 abrogates PGC fate, but this can be rescued by ectopic expression of *SOX17* alone, even in the absence of cytokines, which indicates its pivotal role in hPGC fate (Irie et al., 2015). BLIMP1, which is expressed downstream of SOX17, apparently represses mesendoderm genes, which are expressed in the BLIMP1 mutant cells in response to the cytokines in the medium, including BMP4/BMP2.

CD38, a novel cell surface marker of human germline, is shared by a seminoma cell line, as well as by gonadal hPGCs. Thus, CD38 and tissue non-specific alkaline phosphatase can be used as markers of hPGCLCs for studies using any iPSCs without reporters. CD38 might also be useful to distinguishing between seminomas from embryonal carcinoma cells in human germ cell tumors. Germ cell tumors are thought to arise from blocked PGCs or gonocytes because they develop as carcinomas in situ, from which seminomas and embryonal carcinomas (ECS) can develop (de Jong et al., 2008). Seminomas have properties that resemble early human germ cells, whereas ECS resemble pluripotent stem cells. Thus, seminomas show expression of CD38 and SOX17 whereas embryonal carcinomas show expression of SOX2 and CD30. Seminomas can give rise to embryonal carcinoma cells in germ cell tumors. Based on this observation, it will be of interest to determine if pluripotent embryonic germ cells (hEGCs) can be derived from hPGCs, which could advance knowledge of the relationship between germ cells and pluripotent stem cells.

### Resetting the Epigenome for Totipotency and Development

The human genome is extensively reprogrammed in the germline and during preimplantation development (Tang et al., 2015; Guo et al., 2014; Smith et al., 2014). Epigenetic reprogramming in preimplantation embryo resets the epigenome for naive pluripotency (Takashima et al., 2014), whereas reprogramming in primordial germ cells is more comprehensive than in early embryos, and includes erasure of imprints and potentially epimutations, which restores full germline potency for the transmission of genetic and epigenetic information (Tang et al., 2015). Although there are a number of histone modifications that occur in the early germline, global erasure of DNA methylation to a basal level (to ∼5%) is perhaps the most significant and a unique characteristic of the early germline.

Studies on mouse and human germline reveal some common features as well as differences in the underlying mechanism of DNA demethylation. In both instances, their regulatory network for PGC specification, also acts as the reset switch for the epigenome. In mouse, BLIMP1-PRDM14 are the key factors for the re-set switch, resulting in basal levels of 5-methylcytosine (5mC) (∼2%–3%) in embryonic day 12.5–13.5 mPGCs (Magnúsdóttir et al., 2013; Hackett et al., 2013). Among the targets of BLIMP1-PRDM14 in mouse are DNMT3B, a de novo DNA methylation methyltransferase, and UHRF1, which are repressed by the network. Their repression promotes DNA replication coupled loss of 5mC. Repression of UHRF1 affects maintenance DNA methylation. There are two UHRF1 promoters; one is bound by PRDM14 and the second by BLIMP1 to ensure complete suppression of this genes. Because mouse ESCs express PRDM14 but not BLIMP1, this explains maintenance of UHRF1 expression in ESCs. This partly explains why the erasure of 5mC in mESCs does not reach the low levels seen in mPGCs. Although further work is needed on human PGCs, it is likely that SOX17-BLIMP1 plays a pivotal role in initiating resetting of the epigenome in the human germline, but the involvement of PRDM14 in hPGCs remains unclear (Tang et al., 2015). What is clear is that the enzymes involved in DNA methylation are also repressed in the human germline, but the precise mechanism of their repression is unknown.

The additional mechanism contributing to global DNA demethylation are the enzymes TET1 and TET2; TET1 in particular is highly upregulated in nascent hPGCs (Hackett et al., 2013; Tang et al., 2015). These enzymes convert 5mC to 5hydroxymethylacytosine (5hmC); the latter is also apparently lost through DNA replication-coupled dilution. Additional mechanism may also contribute to DNA demethylation, possibly including base excision repair that could actively excise 5mC (Hajkova et al., 2010). Thus, there are parallel redundant mechanisms that contribute to the comprehensive erasure of 5mC in the early germline. In humans, DNA methylation reaches basal levels in hPGCs during week (Wk) 7–9 of gestation (∼5%).

The global DNA demethylation seen in the germline also accounts for the erasure of genomic imprints during Wk7–Wk9 in hPGCs, before their re-establishment later during gametogenesis, and transmission at fertilization (Tang et al., 2015). Thereafter, imprints are retained and inherited by somatic tissues and they persist into adulthood. Imprinted genes are known to have diverse functions, including growth, metabolism, and behavior, as well as regulation of stem cells and cancers (Lee et al., 2015). Mutations in imprinted genes also account for diverse human diseases, such as Beckwith-Wiedemann and Prader Willi-Angelman syndrome. The imprints need to be erased in primordial germ cells before new imprints that take the form of DNA methylation of imprinting control regions can occur during oogenesis and spermatogenesis. These epigenetic marks that regulate expression of imprinted genes from embryos to adulthood represent an unequivocal example of inheritance of epigenetic information from germline, which is critical for mammalian development.

DNA methylation has a critical role in the repression of TEs; more than half of the human genome is made up of TEs (Zamudio and Bourc’his, 2010). The majority of TEs undergo DNA demethylation in the germline, although the evolutionarily young and active TEs retain partial methylation, suggesting that additional mechanism such as histone modification H3K9me3 might repress TE activity (Tang et al., 2015). A primary mechanism for the repression of TEs involves piRNAs in the mouse male germline. Little is yet known about piRNA biosynthesis in human germline except that many of the genes involved in this pathway are expressed in both male and female hPGCs during Wk7–Wk9, which merits further investigation. Some of the KRAB-ZFP/KAP1 genes that are activated in hPGCs might also have a role in the repression of some TEs (Tang et al., 2015).

### Transgenerational Epigenetic Inheritance

The mechanisms of transgenerational epigenetic inheritance and their consequences are being investigated (Radford et al., 2014; Heard and Martienssen, 2014). Despite comprehensive hypomethylation of the hPGC genome, there are loci that retain significant levels of DNA demethylation in both mouse and human (Hackett et al., 2013; Seisenberger et al., 2012; Tang et al., 2015). While many such “escapees” in hPGCs that retain significant DNA methylation are associated with repeats, there are ∼10% that are repeat-free. Such regions are located at enhancers, CGI, promoters and within gene bodies. Analysis shows that many of these genes with escapees regions are expressed in brain and during neural development. Comparison of this group of genes with the NHGRI GWAS catalog indicates their association with diseases, including obesity-related traits, schizophrenia, and multiple sclerosis. Furthermore, H3K9me3 is the key repressive histone modification epigenetic mark associated with this group of repeat-rich and repeat-poor escapees in selected somatic cell types (Tang et al., 2015). These regions can potentially be targeted by KRAB-ZFP/KAP1 repressive complex, and therefore prone to silencing through heterochromatinization. Motif analysis for two members of KRAB-ZFP/KAF1 family members showed enrichment for repeat-rich escapees and also a moderate enrichment for repeat-poor escapees. Evidence suggests that the repeat-poor escapees retain partial methylation subsequently in the inner cell mass of preimplantation embryos, confirming that they can withstand both waves of erasure of DNA methylation, in hPGCs and early embryos, indicating their potential for transgenerational epigenetic inheritance. These genes are biased toward brain- and growth-related functions and they are therefore candidates for investigations on their potential for epigenetic inheritance. Some escapee loci are shared between mouse and human (Tang et al., 2015). These loci might respond to environmental factors and confer phenotypic plasticity in different tissues.

### Perspective

Recent advances in studies on human germline have elucidated the mechanism of hPGC specification (Irie et al., 2015). There are fundamental differences in the transcriptional regulatory network for PGC specification between mice and human that may be due to differences in their pluripotent states as well as in their postimplantation development. It is important, therefore, to explore non-rodent models for a comprehensive understanding of how this affects PGC specification and other early cell fate decisions. The regulatory network for PGC specification, which includes SOX17-BLIMP1, also acts as a re-set switch for the epigenome, leading to a comprehensive erasure of DNA methylation in hPGCs that restores full germline potency (Tang et al., 2015). This is also the time when the expression of transposable elements is most likely, which requires host defense mechanisms to regulate their activity. A key area of investigation is the apparent transgenerational inheritance of environmentally induced epigenetic information through the germline. Detection of loci that are resistant to reprogramming in the germline provides candidates for investigation for the mechanism that confers protection from erasure and for their potential roles in phenotypic plasticity in human development and disease.
